# High-efficacy global optimization of antenna structures by means of simplex-based predictors

**DOI:** 10.1038/s41598-023-44023-8

**Published:** 2023-10-10

**Authors:** Slawomir Koziel, Anna Pietrenko-Dabrowska

**Affiliations:** 1 Engineering Optimization & Modeling Center, Reykjavik University, 102 Reykjavík, Iceland; 2 Faculty of Electronics, Telecommunications and Informatics, Gdansk University of Technology, 80-233 Gdańsk, Poland

**Keywords:** Electrical and electronic engineering, Computational science

## Abstract

Design of modern antenna systems has become highly dependent on computational tools, especially full-wave electromagnetic (EM) simulation models. EM analysis is capable of yielding accurate representation of antenna characteristics at the expense of considerable evaluation time. Consequently, execution of simulation-driven design procedures (optimization, statistical analysis, multi-criterial design) is severely hindered by the accumulated cost of multiple antenna evaluations. This problem is especially pronounced in the case of global search, frequently performed using nature-inspired algorithms, known for poor computational efficiency. At the same time, global optimization is often required, either due to multimodality of the design task or the lack of sufficiently good starting point. A workaround is to combine metaheuristics with surrogate modeling methods, yet a construction of reliable metamodels over broad ranges of antenna parameters is challenging. This work introduces a novel procedure for global optimization of antenna structures. Our methodology involves a simplex-based automated search performed at the level of approximated operating and performance figures of the structure at hand. The presented approach capitalizes on weakly-nonlinear dependence between the operating figures and antenna geometry parameters, as well as computationally cheap design updates, only requiring a single EM analysis per iteration. Formal convergence of the algorithm is guaranteed by implementing the automated decision-making procedure for reducing the simplex size upon detecting the lack of objective function improvement. The global optimization stage is succeeded by gradient-based parameter refinement. The proposed procedure has been validated using four microstrip antenna structures. Multiple independent runs and statistical analysis of the results have been carried out in order to corroborate global search capability. Satisfactory outcome obtained for all instances, and low average computational cost of only 120 EM antenna simulations, demonstrate superior efficacy of our algorithm, also in comparison with both local optimizers and nature-inspired procedures.

## Introduction

Numerous challenges exist in the design of antenna systems. The major difficulties stem from continuously growing performance demands, driven by the demands of the various areas such as wireless communications^1^ including emerging 5G^2^ and 6G technologies, internet of things (IoT)^3^, automotive radars^4^, space communications^5^, energy harvesting^6^, or medical imaging^7^. These and other applications require specific functionalities (MIMO operation^8^, broadband^9^ and multi-band operation^10^, reconfigurability^11^, high gain^12^, circular polarization^13^, etc.). Meeting these requirements encourages the development of geometrically complex structures that contain additional components incorporated to improve the electrical and field parameters, or enable miniaturization (slots^14^, stubs^15^, shorting pins^16^, custom radiator shapes^17^, defected ground structures^18^, stepped impedance^19^ or tapered feed lines^20^, etc.). Needless to say, a reliable rendition of characteristics of topologically-complex antennas necessitates full-wave electromagnetic (EM) simulation.

Geometrically involved antenna structures are typically described by rather large number of parameters. Identifying the best possible design requires meticulous tuning of all variables, and, as mentioned before, should be performed at the accuracy of EM-simulation models as alternative representations (equivalent networks, analytical descriptions) are either unavailable or grossly inaccurate, and can rarely be parameterized. However, EM-driven optimization tends to be CPU-intensive. Even local (gradient-based) algorithms^21^ may entail as many as a few hundreds of EM analyses, whereas global procedures^22–25^ are incomparably more expensive. Nevertheless, the need for global search arises increasingly frequently in the design of modern antenna systems. Examples include inherently multimodal tasks such as design optimization of frequency-selective surfaces^26^, or coding metasurfaces^27^, size reduction under electrical/field performance constraints^28^, as well as pattern synthesis of array antennas^29,30^. Another case is the lack of sufficiently good initial design^31^. This often occurs for antennas incorporating various geometrical modifications and additional components (stubs, slots, shorting pins, etc.), introduced to enable specific functionalities or miniaturization^32,33^.

Global optimization has been long dominated by nature-inspired algorithms^34–42^. Their history started in 1980s with the development of several fundamental methods such as genetic algorithms (GAs)^43^, evolutionary algorithms (EAs)^44^, genetic programming^45^, ant systems^46^, although evolutionary strategies (ES)^47^ have been proposed for continuous optimization as early as in late 1960s, and can be considered as belonging to the same category. The years 1990s witnessed other significant advancements, specifically, particle swarm optimization (PSO)^48^, as well as differential evolution (DE)^49^, both being popular and widely used until this day. The last decade or so brought numerous new methods, e.g., harmony search^50^, firefly algorithm^51^, grey wolf optimization^52^, and many others^29,53–55^. It seems that despite fancy-looking names (e.g., bacteria foraging optimization^56^, eagle strategy^57^, invasive weed optimization^58^, the list goes on), vast majority of recent techniques employ similar operating principles^59^, and the actual improvements are rather minor over prior methods. In most cases, global search capability is enabled by exchanging information between the elements (individuals, agents, particles, etc.) of the population (swarm, pack, etc.) using appropriate operators^60^, and, in some cases, producing new information through stochastic procedures (e.g., mutation^61^). Implementation of nature-inspired methods is straightforward, with the generic operating flow being almost identical for most procedures^62^. Their most serious disadvantage is poor computational efficiency. A one-time algorithm run may involve a few thousands (or even many thousands) of objective function evaluations. Understandably, this is the major bottleneck for nature-inspired global optimization of antennas whenever the structure at hand is to be evaluated through EM analysis: the entailed CPU cost is simply prohibitive.

Given the aforementioned setbacks, direct application of nature-inspired methods in antenna design is only possible if the underlying merit function is computationally inexpensive (e.g., array factor models utilized for radiation pattern optimization^25,63^), or full-wave simulation is sufficiently fast (only possible for simple components at relatively coarse discretization levels). Another option is parallel computing, however, this is subject to available resources (including software licensing). Nowadays, an increasing attention has been directed towards surrogate modelling methods as acceleration vessels^64–68^. Some of popular techniques include kriging interpolation^69^, and Gaussian Process Regression (GPR)^70^, or artificial neural networks (ANNs)^71^. As a construction of globally accurate model is hardly possible for real-world antennas except simple structures described by a few parameters, surrogates are typically constructed in an iterative manner, based on the EM-simulation data accumulated in the course of the optimization process. The new data samples are allocated using appropriate infill strategies, which may be oriented towards exploitation of the parameter space, its exploration, or combination thereof^72^. In a broader context, acceleration techniques may also involve machine learning methods^73,74^, supplemented by sequential design of experiment strategies^75^. Another possibility is design space pre-screening by means of fast surrogates or decreased-fidelity simulation models^76^.

In antenna modeling, construction of surrogate models faces numerous challenges due to several factors, including dimensionality-related issues and response nonlinearity. Therefore, surrogate-based frameworks are typically demonstrated for restricted search spaces (low dimensionality, narrow parameter ranges)^77–79^. Mitigation of these difficulties has been offered by constrained modelling methods^80–83^, where the metamodel is only constructed in small (volume-wise) regions containing high-quality designs^83^. This does not formally limit the ranges of geometry and/or operational variables the surrogate covers because of exploiting parameter correlations within the optimum-design manifolds, yet permits constructing accurate models using reduced-size data sets^82^, also in variable-fidelity setups^84^. Constrained modelling methods have been applied to accelerate multi-criterial design^85^, and uncertainty quantification^86^. A methodologically distinct approach to expediting EM-based optimization procedures involves response feature technology^87,88^, where the design task is formulated from the standpoint of characteristic points extracted from the system outputs (e.g., antenna resonances, frequencies pertinent to specific levels of reflection or gain responses, etc.). Close-to-linear dependance of the feature points on geometry parameters leads to accelerated convergence of optimization algorithms^89^, or reduced cost of training data acquisition in surrogate modelling^90^.

In this work, we introduce a novel procedure for low-cost quasi-global parameter tuning of antenna structures. The presented approach employs a simplex-based search carried out at the level of operating and performance figures of the system at hand, i.e., the problem-specific knowledge extracted from EM-simulated data. This allows for construction of fast predictor that generates subsequent candidate designs using an automated decision-making procedure which capitalizes on weakly-nonlinear dependence between the operating figures and antenna geometry parameters, similarly as in the response feature frameworks^87^. An automated rendition of new design points is computationally efficient, and requires a single EM analysis per iteration. At the same time, the algorithm incorporates a mechanism for reducing the simplex size upon detecting the lack of objective function improvement, which guarantees a formal convergence of the optimization procedure. The final design is identified by gradient-based parameter tuning that follows the global search stage. The developed framework has been validated using four microstrip antennas, and benchmarked against multiple-start local search as well as nature-inspired optimization. The results demonstrate consistently superior performance of our technique as well as its computational efficiency, with the CPU cost of the optimization process being as low as 120 full-wave simulations on average. The latter is comparable to local algorithms, and significantly lower than for population-based routines.

The originality as well as technical contributions of this work can be outlined as follows: (i) development of the globalized antenna optimization framework involving automated large-scale simplex-based search and feature-like operating parameter approximation, (ii) incorporation of mechanisms to ensure convergence of the optimization process, (iii) implementation of the algorithmic framework including global search stage followed by rapid local tuning, (iv) demonstrating the efficiency of the presented methodology when applied to solving global antenna optimization tasks while maintaining computational cost of the order comparable to the typical expenditures associated with a local search.

## Global optimization of antennas using simplex-based predictors

In this section, we delineate in detail the proposed algorithmic procedure for global optimization of antenna structures. The fundamental tools employed here are simplex-based predictors defined at the level of operating and performance parameters of the antenna at hand, extracted from EM simulation data thereof. The global search step incorporating them allows for identifying—at low computational cost—decent initial designs for further local tuning. The remainder of this section is organized as follows. "Antenna optimization. Problem formulation" section formulates the antenna design problem as a constrained minimization task. "Simplex-based predictors" section introduces simplex-based models, whereas "Global search by means of simplex-based predictors" section discusses their incorporation into a global optimization stage. The procedure for final parameter tuning is outlined in "Local tuning using gradient-based trust-region search" section. "Optimization procedure" section sums up the entire optimization flow.

### Antenna optimization. Problem formulation

**Figure 1:**
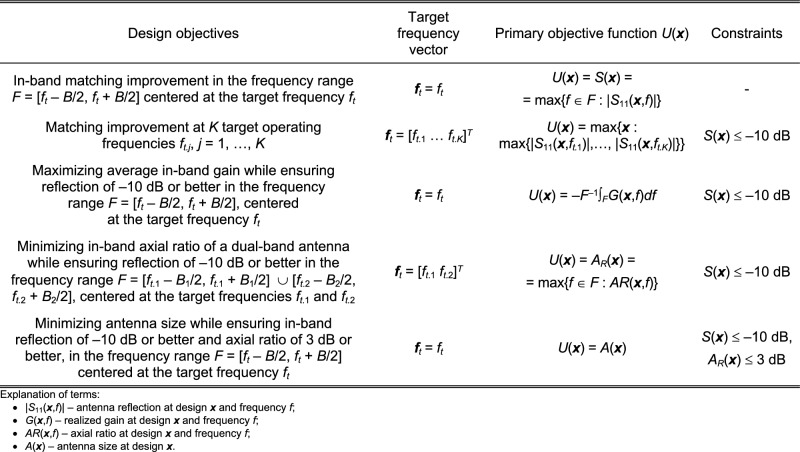
Examples of antenna design optimization scenarios.

Various formulations of antenna optimization task are conceivable, depending on the design goals, constraints, the number and type of characteristics to undergo adjustments, etc. Here, we assume a generic scalar formulation, in which the optimal solution x* is found as

1 $${\varvec{x}}^{*} = \arg \mathop {\min }\limits_{{\varvec{x}}} U({\varvec{x}},{\varvec{f}}_{t} )$$

where *U* is a merit function, and ***f***_*t*_ = [*f*_*t*.1_ … *f*_*t*.*K*_]^*T*^ is a vector of the intended operating frequencies, considered for a general case of a *K*-band antenna. The vector ***x*** = [*x*_1_ … *x*_*n*_]^*T*^ denotes adjustable (usually geometry) parameters of the structure at hand. The problem (1) may be subject to inequality constraints *g*_*k*_(***x***) ≤ 0, *k* = 1, …, *n*_*g*_, and equality constraints *h*_*k*_(***x***) = 0, *k* = 1, …, *n*_*h*_. Figure 1 provides a few examples of typical optimization tasks along with the corresponding objective functions, constraints, and target frequency vectors. The reason for distinguishing the operating frequencies is that appropriate allocation thereof is the major challenge in global optimization of antenna structures, whereas the algorithm proposed in the remaining part of this section is largely based on enforcing required allocation of those frequencies, before proceeding to the local tuning phase.

The treatment of constraints requires a separate note as it presents a challenge on its own. In particular, majority of constraints are expensive, i.e., their evaluation is based on EM analysis results. A convenient handling thereof is an implicit one, by means of a penalty function approach^91^, which is assumed in this work. More specifically, the original problem (1) is reformulated as^91^

2 $${\varvec{x}}^{*} = \arg \mathop {\min }\limits_{{\varvec{x}}} U_{P} ({\varvec{x}})$$

with

3 $$U_{P} ({\varvec{x}}) = U({\varvec{x}}) + \sum\nolimits_{k = 1}^{{n_{g} + n_{h} }} {\beta_{k} c_{k} ({\varvec{x}})}$$

The penalty functions *c*_*k*_(***x***) quantify constraints’ violations, whereas *β*_*k*_ > 0 are the penalty factors. It is convenient to define the functions *c*_*k*_(***x***) to measure relative constraint violations with respect to the assumed threshold, e.g., *c*_*k*_(***x***) = [(*S*(***x***) + 10)/10]^2^ for impedance matching (cf. Fig. 1 for the explanation of *S*(***x***)). The use of the second power [.]^2^ serves two purposes: (i) ensuring that *U*_*P*_ at the feasible region boundary is smooth, and (ii) providing a leeway for small violations.

### Simplex-based predictors

**Figure 2:**
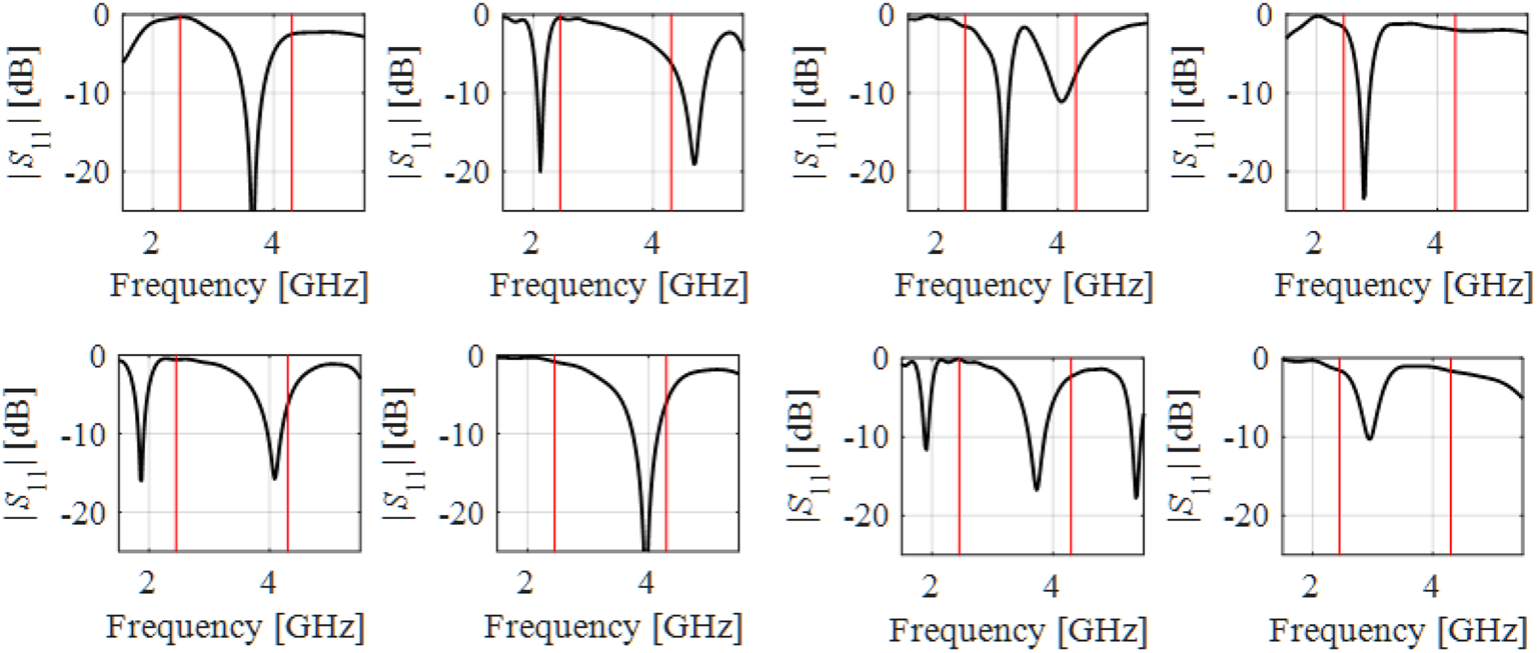
Exemplary reflection characteristics of a dual-band antenna at random designs belonging to the assumed design space with the intended operating frequencies 2.45 GHz and 4.3 GHz indicated using vertical lines. Local search initiated from over a half of the presented designs would fail as a result of a considerable misalignment of the target and existing antenna resonances.

Attaining the best achievable performance of antenna structures requires meticulous and simultaneous tuning of all relevant geometry parameters, which is a challenging endeavour due to high computational expenses entailed by repetitive EM analyses involved in the process. As explained in "Introduction" section, global search is required in many cases, either due to inherent multimodality of the problem, or the lack of sufficiently good initial design. EM-based antenna miniaturization, dimension scaling of multi-band antenna structures for new operational frequencies, or radiation pattern synthesis of array antennas, are just a few examples. Global optimization requires exploring the parameter space in its entirety, which is impeded by nonlinearity of antenna responses and significant relocations of operating frequencies across the space. To illustrate the issue, Fig. 2 presents responses of an exemplary dual-band antenna evaluated at a number of random parameter vectors. Clearly, local search initiated from the majority of these points would fail to place the antenna resonances at the intended values (marked red in the plots of Fig. 2). At the same time, rendering accurate surrogate for this sort of characteristics requires a large number of training data points, acquisition of which may be prohibitive in computational terms.

**Figure 3:**
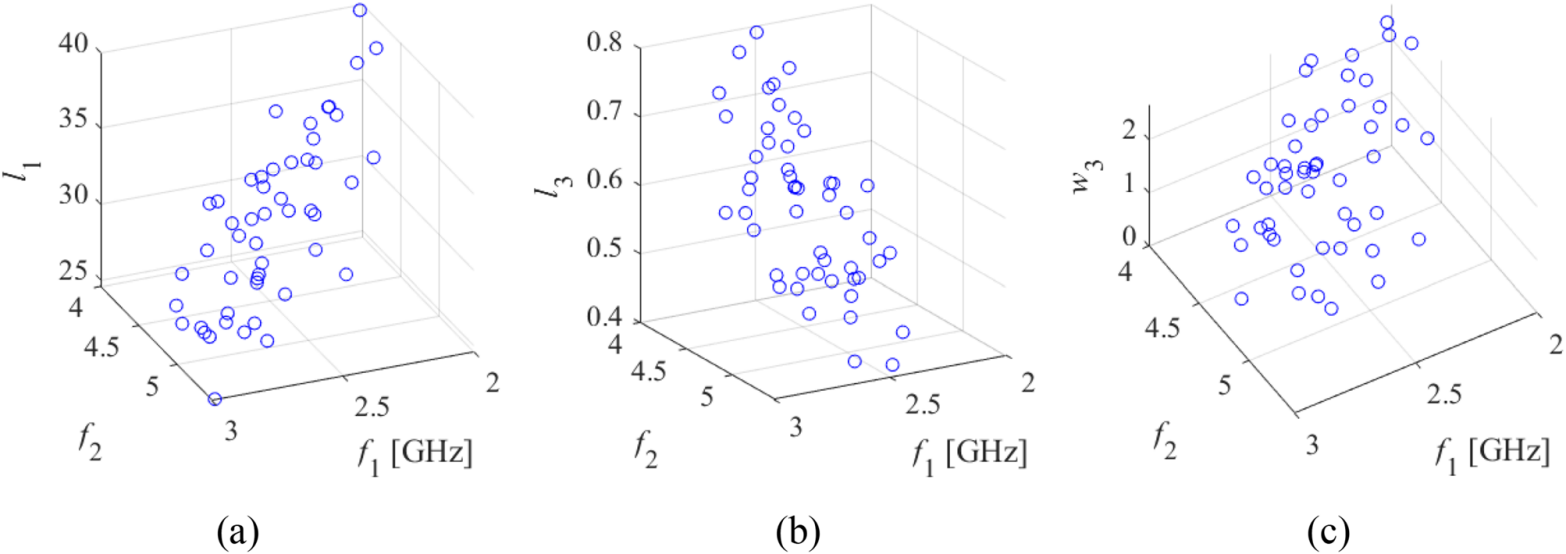
Relationship between operating parameters (here, resonant frequencies *f*_1_ and *f*_2_) and selected geometry parameters of the antenna of Fig. 2a, obtained for a number of random trial points. To create the plots, only those points were chosen, for which the corresponding characteristics show clearly visible resonances allocated within the ranges of interest, as shown in axis description. Clear patterns are visible even though the trial points were not optimized whatsoever.

The situation changes dramatically when the problem is considered from the perspective of operating parameters of the structure. Figure 3 shows the relationships between the resonant frequencies *f*_1_ and *f*_2_ of the antenna of Fig. 2a and its selected geometry parameters. The plots were obtained from a number of random trial points. Despite the fact that the parameter vectors are not optimized, clear patterns can be observed, along with well pronounced correlations between the spaces of operating and geometry parameters. As demonstrated in the literature, especially in the context of feature-based modelling and optimization^87–90^, this sort of relationship is rather universal.

Having in mind Fig. 3, in this work, the problem of global optimization of antennas is approached from the standpoint of the relationships illustrated therein. Towards this end, problem-specific knowledge embedded in antenna responses has to be employed. Due to geometrical simplicity of the said relations, it is sufficient—for the purpose of globalized optimization—to construct relatively simple surrogate models (predictors), defined at the level of operating and performance figures rather than directly geometry parameters and antenna characteristics in their entirety. In order to account for all *n* directions within the parameter space, the model has to involve at least *n* + 1 distinct vectors ***x***^(*j*)^. The simplest structure that enables this, assuming almost arbitrary point allocation, is a simplex. In the following, we formally define the simplex-based predictor employed in this paper and explain how it is used in the search process.

**Figure 4:**
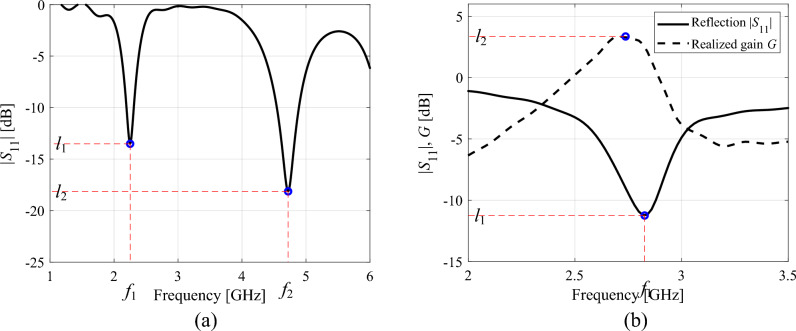
Examples of the operating and performance parameters: (**a**) exemplary dual-band antenna with the operating figure vector consisting of antenna resonant frequencies ***f*** = [*f*_1_ *f*_2_]^*T*^, and the performance figure vector consisting of reflection levels at the centre frequencies ***l*** = [*l*_1_ *l*_2_]^*T*^; (**b**) exemplary quasi-Yagi antenna with the operating figure vector containing the centre frequency ***f*** = *f*_1_, and the performance figure vector containing reflection level at *f*_1_ and maximum realized gain value *l*_2_, i.e., ***l*** = [*l*_1_ *l*_2_]^*T*^.

Before proceeding further, let us introduce the notion of operating figure vectors ***f*** = [*f*_1_ … *f*_*N*_]^*T*^, and performance figure vectors ***l*** = [*l*_1_ … *l*_*M*_]^*T*^. The first will be used to denote the target quantities (e.g., centre frequency for a narrow-band antenna, bandwidth, power division ratio for a coupling structure, or even material parameters, e.g., substrate permittivity the device in fabricated on). For a multi-band antenna with target operating frequency vector ***f***_*t*_ discussed earlier, the vector ***f*** would coincide with ***f***_*t*_. The performance figure vector ***l*** contains quantities used to determine design quality (other than those already allocated in the vector ***f***). These may include reflection levels at the antenna resonances (or over specific frequency ranges), the value of gain or axial ratio at the centre frequency, side lobe levels, etc. Figure 4 shows examples of operating and performance figure vectors for an exemplary dual-band and quasi-Yagi antennas.

Let *X* be the space of design parameters demarked by the lower and upper bounds for antenna parameters. Furthermore, let ***f***_*L*_ = [*f*_*L*.1_ … *f*_*L.N*_]^*T*^ and ***f***_*U*_ = [*f*_*U*.1_ … *f*_*U.N*_]^*T*^ be the acceptance bounds for the operating figure vector, meaning that we are only interested in vectors that satisfy *f*_*L.j*_ ≤ *f*_*j*_ ≤ *f*_*U.j*_, *j* = 1, …, *N*. Similarly, let ***l***_*L*_ = [*l*_*L*.1_ … *l*_*L.M*_]^*T*^ and ***l***_*U*_ = [*l*_*U*.1_ … *l*_*U.M*_]^*T*^ be the acceptance bounds for the performance figure vector; we want to maintain *l*_*L.j*_ ≤ *l*_*j*_ ≤ *l*_*U.j*_, *j* = 1, …, *M*. Some of the *l*_*L.j*_ may be equal to –∞, meaning that there is no lower bound for *l*_*j*_. We may also have *l*_*U.j*_ = ∞, meaning that there is no upper bound for *l*_*j*_.

**Figure 5:**
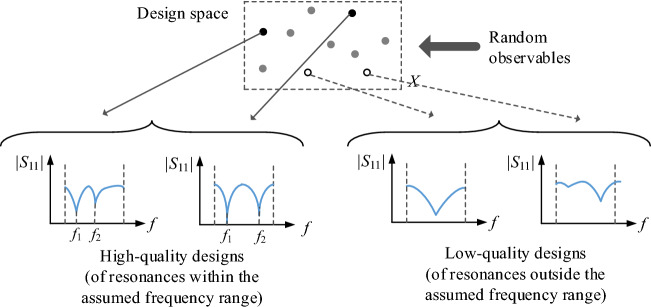
Generating random trial points for the purpose of simplex-based predictor construction. Illustration based on the antenna of Fig. 2a. The operating parameters are resonant frequencies *f*_1_ and *f*_2_. Only parameter vectors with the corresponding antenna responses featuring clearly visible resonances allocated within the prescribed ranges are accepted to become the basis to build the predictor.

Let ***x***^(*j*)^ = [*x*_1_^(*j*)^ … *x*_*n*_^(*j*)^]^*T*^, *j* = 0, …, *n*, be *n* + 1 affinely independent points in *X*, ***f***^(*j*)^ = ***f***(***x***^(*j*)^) = [*f*_1_^(*j*)^ … *f*_*N*_^(*j*)^]^*T*^ be the corresponding operating figure vectors, and ***l***^(*j*)^ = ***l***(***x***^(*j*)^) = [*l*_1_^(*j*)^ … *l*_*K*_^(*j*)^]^*T*^ be the performance figure vectors. The vectors ***x***^(*j*)^ are obtained to ensure that that ***f***_*L*_ ≤ ***f***^(*j*)^ ≤ ***f***_*U*_, and ***l***_*L*_ ≤ ***l***^(*j*)^ ≤ ***l***_*U*_. In practice, an automated decision-making procedure is employed, in which random vectors are generated sequentially, and only those that satisfy the above conditions (the operating and performance vectors are extracted from EM simulation data), are accepted. The procedure is terminated after *n* + 1 points have been accepted, which are additionally affinely independent. A conceptual illustration of the random sampling procedure has been shown in Fig. 5.

We are now in a position to define the simplex-based predictors. Let ***x*** ∈ *X*. Linear independence of vectors ***x***^(*j*)^ – ***x***^(0)^ implies that we have a unique expansion

4 $${\varvec{x}} = {\varvec{x}}^{(0)} + \sum\limits_{j = 1}^{n} {a_{j} ({\varvec{x}}^{(j)} - {\varvec{x}}^{(0)} )}$$

The expansion coefficient vector ***a*** = [*a*_1_ … *a*_*n*_]^*T*^ can be found as

5 $${\varvec{a}}({\varvec{x}}) = {\varvec{X}}^{ - 1} ({\varvec{x}} - {\varvec{x}}^{(0)} )$$

where ***X*** is a non-singular *n* × *n* matrix defined as

6 $${\varvec{X}} = \left[ {{\varvec{x}}^{(1)} - {\varvec{x}}^{(0)} \;\; \cdots \;\;\;{\varvec{x}}^{(n)} - {\varvec{x}}^{(0)} } \right]$$

The simplex-based models of the operating parameters ***F***(***x***) : *X* → *F*, and performance parameters ***L***(***x***) : *X* → *R*^*M*^, to be used as predictors of the operating and performance vectors over *X* and *R*^*M*^, respectively, are set up with the use of the problem-specific knowledge extracted form the antenna responses. The predictors are defined as

7 $${\varvec{F}}({\varvec{x}}) = {\varvec{f}}^{(0)} + \sum\limits_{j = 1}^{n} {a_{j} } ({\varvec{f}}^{(j)} - {\varvec{f}}^{(0)} ) = {\varvec{f}}^{(0)} + {\varvec{X}}_{f} {\varvec{a}}({\varvec{x}}) = {\varvec{f}}^{(0)} + {\varvec{X}}_{f} {\varvec{X}}^{ - 1} ({\varvec{x}} - {\varvec{x}}^{(0)} )$$

8 $${\varvec{L}}({\varvec{x}}) = {\varvec{l}}^{(0)} + \sum\limits_{j = 1}^{n} {a_{j} } ({\varvec{l}}^{(j)} - {\varvec{l}}^{(0)} ) = {\varvec{l}}^{(0)} + {\varvec{X}}_{l} {\varvec{a}}({\varvec{x}}) = {\varvec{l}}^{(0)} + {\varvec{X}}_{l} {\varvec{X}}^{ - 1} ({\varvec{x}} - {\varvec{x}}^{(0)} )$$

with ***a*** computed as (5), and

9 $${\varvec{X}}_{f} = \left[ {{\varvec{f}}^{(1)} - {\varvec{f}}^{(0)} \;\;\; \cdots \;\;\;{\varvec{f}}^{(n)} - {\varvec{f}}^{(0)} } \right]$$

10 $${\varvec{X}}_{l} = \left[ {{\varvec{l}}^{(1)} - {\varvec{l}}^{(0)} \;\;\; \cdots \;\;\;{\varvec{l}}^{(n)} - {\varvec{l}}^{(0)} } \right]$$

The models ***F***(***x***) and ***L***(***x***) will be employed to yield predictions about the operating and performance vectors over the parameter space *X* as explained in "Global search by means of simplex-based predictors" section.

### Global search by means of simplex-based predictors

The models ***F***(***x***) and ***L***(***x***) defined in "Simplex-based predictors" section are the basis for performing the global search step of the proposed optimization algorithm. As explained earlier (cf. "Antenna optimization. Problem formulation" section and Fig. 3), the dependence between the operating and performance vectors ***f*** and ***l***, and the antenna geometry parameters is weakly nonlinear (especially for ***f***). This means that the models (7) and (8) are likely to act as reliable predictors of antenna performance over *X*, particularly within the simplex. 

#### Design quality assessment

Evaluation of design quality is an important consideration in any optimization process. Let *U*_*F*_ be the objective function defined to compute the design quality by taking into account the vectors ***f***(***x***) and ***l***(***x***). Therein the priority is given to reducing the distance between the existent operating vector and the target ***f***_*t*_. Note that we use the same symbol ***f***_*t*_ to denote the target operating figure vector and the target operating frequency vector of "Antenna optimization. Problem formulation" section, which is for notational simplicity but also because in all examples considered in "Demonstration case studies" section, both vectors coincide. The function *U*_*F*_ is defined as

11 $$U_{F} ({\varvec{x}}) = U({\varvec{f}}({\varvec{x}}),{\varvec{l}}({\varvec{x}})) = U_{L} ({\varvec{l}}({\varvec{x}})) + \beta_{F} ||{\varvec{f}}({\varvec{x}}) - {\varvec{f}}_{t} ||^{2}$$

Here, *U*_*L*_ is the merit function defined similarly as the function *U* of "Antenna optimization. Problem formulation" section, however, computed based on ***l***(***x***) rather than the entire antenna characteristics. For example if ***l***(***x***) = [*l*_1_ …, *l*_*M*_]^*T*^ represent reflection levels of a *M*-band antenna, and the aim is to improve the matching at all operational frequencies *f*_1_ through *f*_*M*_, then we have *U*_*L*_(***l***(***x***)) = max{*l*_1_,…,*l*_*M*_}. If we intend to increase the antenna gain at the operating frequency *f*_1_, with the performance vector defined as for the quasi-Yagi antenna of Fig. 4b, i.e., ***l*** = [*l*_1_* l*_2_]^*T*^, with *l*_2_ being the maximum gain, we may define *U*_*L*_(***l***(***x***)) = –*l*_2_. These functions may not be exact equivalents of functions *U*(***x***), especially if certain response levels are to be minimized/maximized over bandwidths, but at this stage we are mainly focused on enforcing ***f***(***x***) → ***f***_*t*_. The latter is achieved by the second (penalty) term in (11), where *β*_*F*_ is a penalty factor.

#### Global search: automated simplex update

The global search stage is an iterative process, in which the fundamental step is minimization of the function *U*_*F*_ using the simplex-based predictors ***F***(***x***) and ***L***(***x***). The candidate design is produced as

12 $${\varvec{x}}_{tmp} = \arg \mathop {\min }\limits_{{{\varvec{x}} \in X}} U_{F} ({\varvec{F}}({\varvec{x}}),{\varvec{L}}({\varvec{x}}))$$

**Figure 6:**
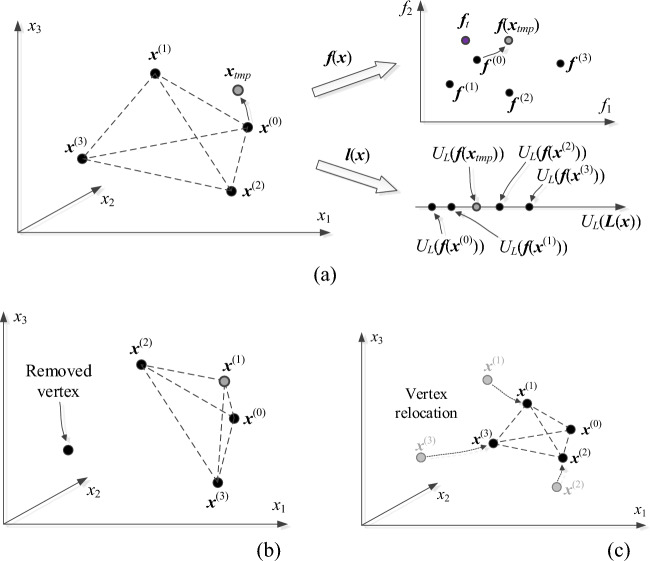
Global search stage by simplex updates: (**a**) exemplary simplex in a three-dimensional design space. The predictions made using the simplex-based models ***F***(***x***) and ***L***(***x***) are validated against actual functions ***f***(***x***) and ***l***(***x***) at the candidate design ***x***_*tmp*_ produced by (12). In the situation shown in the picture, the candidate design will be accepted due to improving both the antenna performance function *U*_*L*_ and the factor ||***f***(***x***) – ***f***_*t*_||; (**b**) simplex updating for accepted candidate design. In the situation shown, ***x***_*tmp*_ was better than ***x***^(1)^ but not as good as ***x***^(0)^; (**c**) simplex reduction upon rejecting candidate design: all vertices ***x***^(*j*)^, *j* = 1, …, *n*, are moved towards ***x***^(0)^.

The problem is constrained to ensure that the search process is carried out solely inside of the simplex defined by {***x***^(*j*)^}_*j* = 0,…,*n*_ and its small vicinity. The constraints are imposed on the expansion coefficients ***a***(***x***) of (5). We have

13 $$\sum\limits_{j = 1}^{n} {a_{j} = 1}$$

14 $$- \alpha \le a_{j} \le 1 + \alpha ,\;\;j\, = \,{1}, \, \ldots ,n$$

where *α* > 0 is a small real number (e.g., *α* = 0.2). The simplex vertices are ordered with respect to increasing norm ||***f***^(*j*)^ – ***f***_*t*_||, i.e., the best vertex, ***x***^(0)^ is the one that features the smallest value of the mentioned norm. The starting point for (12) is ***x***^(0)^ because this is the design that is the closest to the target in the sense explained earlier. Note that its corresponding expansion vector ***a***(***x***^(0)^) = [0 … 0]^*T*^. Figure 6 shows graphically the concepts related to the simplex-based predictor and generation of the candidate design ***x***_*tmp*_.

The candidate design is accepted assuming that it leads to the overall improvement of the simplex quality, i.e., if

15 $$\left| {\left| {\varvec{f}_{tmp} -\varvec{f}_{t} } \right|} \right| < \,{\text{max}}\left\{ {j\, \in \,\left\{ {0,{ 1}, \, \ldots ,n} \right\}:||\varvec{f}^{(j)} -\varvec{f}_{t} ||} \right\}$$

where ***f***_*tmp*_ = ***f***(***x***_*tmp*_). If this is the case, ***x***_*tmp*_ replaces the worst vertex ***x***^(*jworst*)^, where

16 $$j_{worst} \, = \,{\text{argmax}}\{ j\, \in \,\left\{ {0,{ 1}, \, \ldots ,n} \right\}:\left| {\left| {\varvec{f}^{(j)} -\varvec{f}_{t} } \right|} \right|\}$$

Otherwise, the vector is rejected, and the simplex is reduced towards the best vertex ***x***^(0)^ using the following transformation:

17 $${\varvec{x}}^{(j)} \leftarrow \gamma {\varvec{x}}^{(j)} + (1 - \gamma ){\varvec{x}}^{(0)} ,\;\;{\text{for}}\;\;j\, = \,{1}, \, \ldots ,n$$

Here, *γ* is the reduction factor, normally set up to *γ* = 0.5, meaning that the simplex is reduced by half with ***x***^(0)^ remaining intact. Note that this operation will generally improve the quality of all vertices, i.e., reduce the factors ||***f***^(*j*)^ – ***f***_*t*_||. In "Objective function improvement" section, we prove that under mild assumptions, reduction of the simplex size will eventually lead to generating candidate designs that will be accepted due to reducing ||***f***(***x***_*tmp*_) – ***f***_*t*_||.

The above procedure is continued until we find a design that satisfies the condition

18 $$||{\varvec{f}}({\varvec{x}}_{tmp} ) - {\varvec{f}}_{t} || \le F_{\max }$$

where *F*_max_ is a user-defined threshold. It is set up to ascertain that the optimum design is within the reach of the subsequent local tuning. This means that *F*_max_ has to be equal to a fraction (e.g., no more than half) of the expected antenna operating bandwidths. The value of this parameter is not critical as long it is sufficiently small, e.g., 0.1 or 0.2 GHz for antennas working in the ranges of up to a few GHz or so. Other termination conditions are based on exceeding the assumed computational budget (*N*_*global*_ EM analyzes) or reducing the simplex size *D* = max{*j* ∈ {1,2,…,*n*} : ||***x***^(*j*)^ – ***x***^(0)^||} beyond the user-defined threshold *D*_min_. Both are used to ensure convergence of the optimization process even if the condition (18) is unattainable.

#### Objective function improvement

In this section, we provide a formal proof that sufficient reduction of the simplex size, according to (18) will ensure improvement of the objective function *U*_*F*_ as well as the factor ||***f***(***x***) – ***f***_*t*_|| (both over their values at ***x***^(0)^). The sole assumption is made of the smoothness of the involved functions.

Let functions ***f***(***x***) and ***l***(***x***) be continuously differentiable in *X*. Then, we have

19 $${\varvec{f}}({\varvec{x}}) \approx {\varvec{f}}({\varvec{x}}^{(0)} ) + {\varvec{J}}_{f} ({\varvec{x}}^{(0)} ) \cdot ({\varvec{x}} - {\varvec{x}}^{(0)} )$$

20 $${\varvec{l}}({\varvec{x}}) \approx {\varvec{l}}({\varvec{x}}^{(0)} ) + {\varvec{J}}_{l} ({\varvec{x}}^{(0)} ) \cdot ({\varvec{x}} - {\varvec{x}}^{(0)} )$$

in a sufficiently small vicinity of the vector ***x***^(0)^, where

21 $${\varvec{J}}_{f} ({\varvec{x}}) = \left[ {\begin{array}{*{20}c} {\frac{{\partial f_{1} ({\varvec{x}})}}{{\partial x_{1} }}} & \cdots & {\frac{{\partial f_{1} ({\varvec{x}})}}{{\partial x_{n} }}} \\ \vdots & \ddots & \vdots \\ {\frac{{\partial f_{N} ({\varvec{x}})}}{{\partial x_{1} }}} & \cdots & {\frac{{\partial f_{N} ({\varvec{x}})}}{{\partial x_{n} }}} \\ \end{array} } \right]\;\;\;{\text{and}}\;\;\;{\varvec{J}}_{l} ({\varvec{x}}) = \left[ {\begin{array}{*{20}c} {\frac{{\partial l_{1} ({\varvec{x}})}}{{\partial x_{1} }}} & \cdots & {\frac{{\partial l_{1} ({\varvec{x}})}}{{\partial x_{n} }}} \\ \vdots & \ddots & \vdots \\ {\frac{{\partial l_{M} ({\varvec{x}})}}{{\partial x_{1} }}} & \cdots & {\frac{{\partial l_{M} ({\varvec{x}})}}{{\partial x_{n} }}} \\ \end{array} } \right]$$

By applying (19) and (20) to all simplex vertices, we get

22 $${\varvec{f}}^{(j)} = {\varvec{f}}({\varvec{x}}^{(j)} ) \approx {\varvec{f}}({\varvec{x}}^{(0)} ) + {\varvec{J}}_{f} ({\varvec{x}}^{(0)} ) \cdot ({\varvec{x}}^{(j)} - {\varvec{x}}^{(0)} )$$

23 $${\varvec{l}}^{(j)} = {\varvec{l}}({\varvec{x}}^{(j)} ) \approx {\varvec{l}}({\varvec{x}}^{(0)} ) + {\varvec{J}}_{l} ({\varvec{x}}^{(0)} ) \cdot ({\varvec{x}}^{(j)} - {\varvec{x}}^{(0)} )$$

for *j* = 1, …, *n*. By applying (22) to (9), and (23) to (10), we obtain

24 $${\varvec{X}}_{f} \approx \left[ {{\varvec{J}}_{f} ({\varvec{x}}^{(0)} ) \cdot ({\varvec{x}}^{(1)} - {\varvec{x}}^{(0)} )\;\;\; \cdots \;\;\;{\varvec{J}}_{f} ({\varvec{x}}^{(0)} ) \cdot ({\varvec{x}}^{(n)} - {\varvec{x}}^{(0)} )} \right] = {\varvec{J}}_{f} ({\varvec{x}}^{(0)} ){\varvec{X}}$$

25 $${\varvec{X}}_{l} \approx \left[ {{\varvec{J}}_{l} ({\varvec{x}}^{(0)} ) \cdot ({\varvec{x}}^{(1)} - {\varvec{x}}^{(0)} )\;\;\; \cdots \;\;\;{\varvec{J}}_{l} ({\varvec{x}}^{(0)} ) \cdot ({\varvec{x}}^{(n)} - {\varvec{x}}^{(0)} )} \right] = {\varvec{J}}_{l} ({\varvec{x}}^{(0)} ){\varvec{X}}$$

This results in (cf. (7) and (8))

26 $${\varvec{F}}({\varvec{x}}) \approx {\varvec{f}}^{(0)} + {\varvec{J}}_{f} ({\varvec{x}}^{(0)} ){\varvec{XX}}^{ - 1} ({\varvec{x}} - {\varvec{x}}^{(0)} ) = {\varvec{f}}^{(0)} + {\varvec{J}}_{f} ({\varvec{x}}^{(0)} )({\varvec{x}} - {\varvec{x}}^{(0)} ) \approx {\varvec{f}}({\varvec{x}})$$

and

27 $${\varvec{L}}({\varvec{x}}) \approx {\varvec{l}}^{(0)} + {\varvec{J}}_{l} ({\varvec{x}}^{(0)} ){\varvec{XX}}^{ - 1} ({\varvec{x}} - {\varvec{x}}^{(0)} ) = {\varvec{l}}^{(0)} + {\varvec{J}}_{l} ({\varvec{x}}^{(0)} )({\varvec{x}} - {\varvec{x}}^{(0)} ) \approx {\varvec{l}}({\varvec{x}})$$

in a sufficiently small neighbourhood of ***x***^(0)^, i.e., when the simplex size *D* = max{*j* ∈ {1,2,…,*n*} : ||***x***^(*j*)^ – ***x***^(0)^||} is close to zero. In particular, the Jacobian matrices ***J***_*F*_ and ***J***_*L*_ of ***F*** and ***L*** at ***x***^(0)^, coincide with the respective matrices ***J***_*f*_ and ***J***_*l*_ of ***f*** and ***l***. Consequently, when *D* → 0, the predictions of the simplex-based models (7) and (8) coincide with the predictions of the Taylor models (19) and (20), i.e., the outcome of the simplex updating iteration ***x***_*tmp*_ (cf. (12)) will result in the improvement of the objective function *U*_*F*_ of (11). The latter is implied by the following observation. The first-order expansion model of *U*_*F*_ is given as

28 $$U_{F} ({\varvec{x}}) = U_{F} ({\varvec{f}}({\varvec{x}}),{\varvec{l}}({\varvec{x}})) \approx U_{F} ({\varvec{x}}^{(0)} ) + \left[ {\nabla_{f}^{T} {\varvec{J}}_{f} ({\varvec{x}}^{(0)} ) + \nabla_{l}^{T} {\varvec{J}}_{l} ({\varvec{x}}^{(0)} )} \right]({\varvec{x}} - {\varvec{x}}^{(0)} )$$

where ∇_*f*_ and ∇_*l*_ are the gradients of *U*_*F*_ at ***x***^(0)^. Then, any descent direction ***h***, i.e., such that $[\nabla_{f}^{T} {\varvec{J}}_{f} ({\varvec{x}}^{(0)} ) + \nabla_{l}^{T} {\varvec{J}}_{l} ({\varvec{x}}^{(0)} )]{\varvec{h}} < 0$ is also a descent direction according to the simplex-based models because $[\nabla_{f}^{T} {\varvec{J}}_{f} ({\varvec{x}}^{(0)} ) + \nabla_{l}^{T} {\varvec{J}}_{l} ({\varvec{x}}^{(0)} )] \approx [\nabla_{f}^{T} {\varvec{J}}_{F} ({\varvec{x}}^{(0)} ) + \nabla_{l}^{T} {\varvec{J}}_{L} ({\varvec{x}}^{(0)} )]$. In a similar manner, one can show a reduction of ||***f***(***x***) – ***f***_*t*_||.

### Local tuning using gradient-based trust-region search

Upon finding the design ***x***^(0)^ that is close enough to the optimum (cf. (18)), a local tuning procedure is launched to refine the antenna parameters. Here, it carried out using the trust-region (TR) gradient-based routine with numerical derivatives^92^. The procedure is an iterative one, and it yields a sequence ***x***^(*i*)^, *i* = 0, 1, …, of approximations of the optimal solution x^*^. These designs are rendered by solving

29 $${\varvec{x}}^{(i + 1)} = \arg \mathop {\min }\limits_{{||{\varvec{x}} - {\varvec{x}}^{(i)} || \le d^{(i)} }} U_{L} ({\varvec{x}},{\varvec{f}}_{t} )$$

The objective function *U*_*L*_ is identical to the function *U*_*P*_ of (3), except that it is evaluated based on the first-order linear expansion model ***L***^(*i*)^(***x***,*f*) of antenna characteristics, instead of original, EM-simulated responses. If, for example, we consider the reflection response *S*_11_(***x***,*f*), the linear model becomes

30 $$\varvec{L}^{\left( i \right)} \left( {\varvec{x},f} \right)\, = \,S_{{{11}}} \left( {\varvec{x}^{(i)} ,f} \right)\, + \,\nabla_{S} \left( {\varvec{x}^{(i)} ,f} \right) \cdot \left( {\varvec{x}-\varvec{x}^{(i)} } \right)$$

Finite differentiation is employed to evaluate the gradient in (30). The solution of the task (29) is sought for in a small vicinity of the actual design with its size *d*^(*i*)^ set using the conventional TR rules^92^. The new point ***x***^(*i*+1)^ is accepted if it improves the original (EM-evaluated) merit function, i.e., *U*_*P*_(***x***^(*i*+1)^,***f***_*t*_) < *U*_*P*_(***x***^(*i*)^,***f***_*t*_). Otherwise, it is dismissed and the iteration is re-launched within a smaller *d*^(*i*)^. The CPU cost of constructing the model (30) is equivalent to *n* + 1 EM antenna simulations. In order to diminish this cost, in our implementation, the rank-one Broyden formula^93,94^, is employed instead of finite differentiation, when the search approaches convergence, i.e., ||***x***^(*i*+1)^ – ***x***^(*i*)^||< *M*_*c*_*ε*, where *ε* is the termination threshold, whereas *M*_*c*_ is the multiplication factor, e.g., *M*_*c*_ = 10. The algorithm is terminated either due to the convergence in argument ||***x***^(*i*+1)^ – ***x***^(*i*)^||< *ε*, or a sufficient diminution of the trust region *d*^(*i*)^ < *ε* (whichever occurs first). In our experiments, we assume *ε* = 10^–3^.

### Optimization procedure

Here, we put together all constituent parts of the developed optimization procedure in a form of a pseudocode. The input variables are the following:

- Parameter space *X*;
- Definitions of operating and performance vectors ***f*** and ***l***;
- Target operating parameter vector ***f***_*t*_;
- Merit function *U*_*L*_ (cf. (11)).

The first two inputs depend on the antenna structure to be optimized, whereas the remaining two are governed by the design problem at hand.

The control parameters of our framework are the following:

- *F*_max_—a user-defined threshold for termination of the global search stage taking into account the distance between the target and actual operating parameter vector (cf. (18));
- *α*—search region extension parameter (cf. (14)), normally set to small positive number, e.g., *α* = 0.2;
- *γ*—simplex reduction ratio (cf. (17)), normally set to *γ* = 0.5;
- *D*_min_—termination threshold (minimum simplex size leading to termination of global search stage), normally set to a small fraction (e.g., 1%) of the parameter space size;
- *ε*—termination threshold for local tuning stage, normally set to an assumed resolution of the optimization process, e.g., 10^–3^ (cf. "Local tuning using gradient-based trust-region search" section).

**Figure 7:**
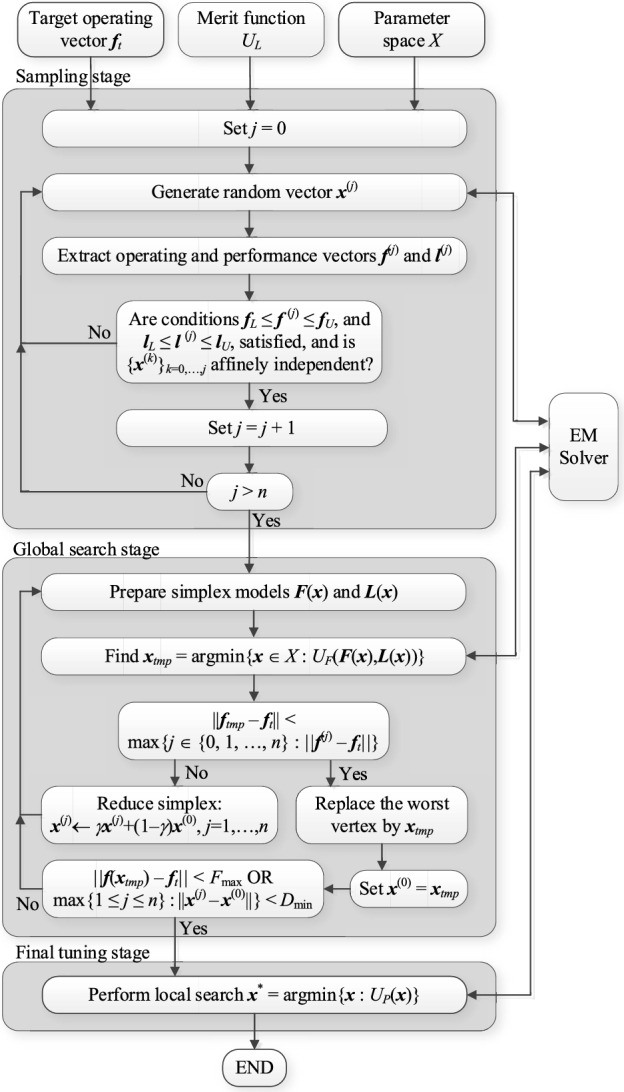
Flowchart of the developed globalized antenna optimization procedure using simplex-based predictors.

Observe that none of the above parameters is critical for the performance of the algorithm, except *F*_max_, which needs to be established as suggested in "Global search: automated simplex update" section, i.e., to a fraction of the expected antenna operating bandwidths. Figure 7 presents a flow diagram of the developed framework.

## Demonstration case studies

This part of the paper discusses validation of the antenna optimization algorithm introduced in the previous section. It is based on four microstrip devices, including dual- and triple-band structures, as well as a quasi-Yagi antenna. The numerical experiments aim to verify the global search capability of out framework, as well as to compare it with benchmark methods, specifically, multiple-start local optimization and nature-inspired routines. The latter is represented by a particle swarm optimizer (PSO)^95^, which is arguably the most widely utilized population-based algorithm nowadays. The primary factors that are of interest are the optimization process reliability, quality of the design produced in the course of optimization, but also computational cost.

In terms of organization, "Verification antenna structures" section provides the relevant data about the verification antenna structures, and the corresponding design problems. The setup of experiments is outlined in "Experimental setup" section, whereas "Results and discussion" section provided the results and their discussion.

### Verification antenna structures

**Figure 8:**
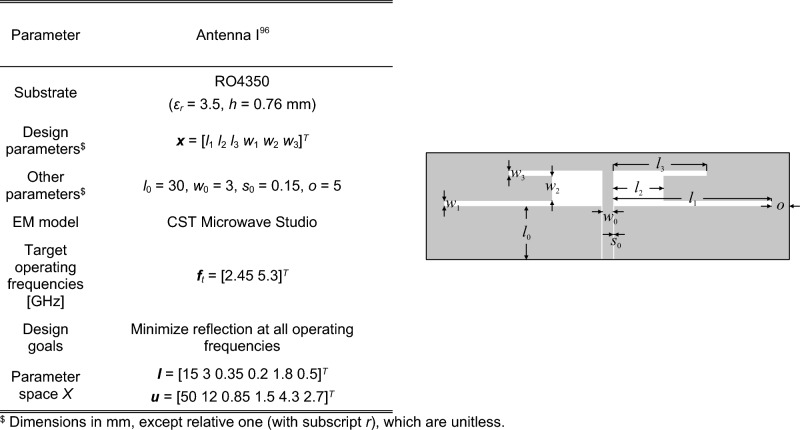
Antenna I^96^: dual-band uniplanar dipole antenna; parameters (left), and antenna geometry (right).

**Figure 9:**
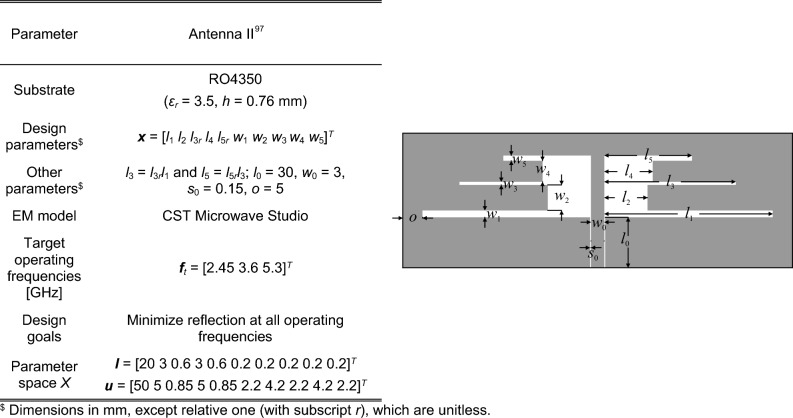
Antenna II^97^: triple-band uniplanar dipole antenna; parameters (left), and antenna geometry (right).

**Figure 10:**
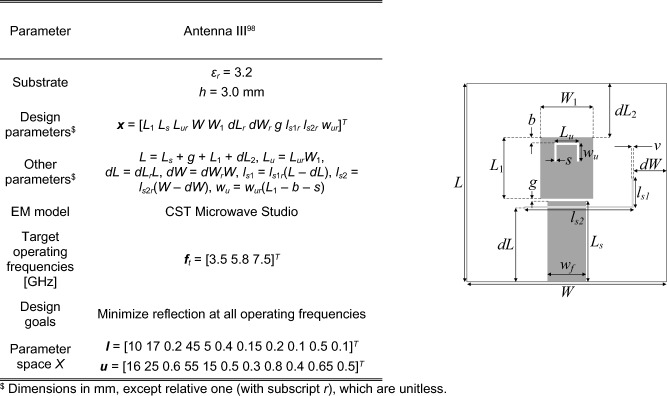
Antenna III^98^: triple-band U-slotted patch antenna with defected ground structure (DSG), the light-shade grey denotes a ground-plane slot; parameters (left), and antenna geometry (right).

**Figure 11:**
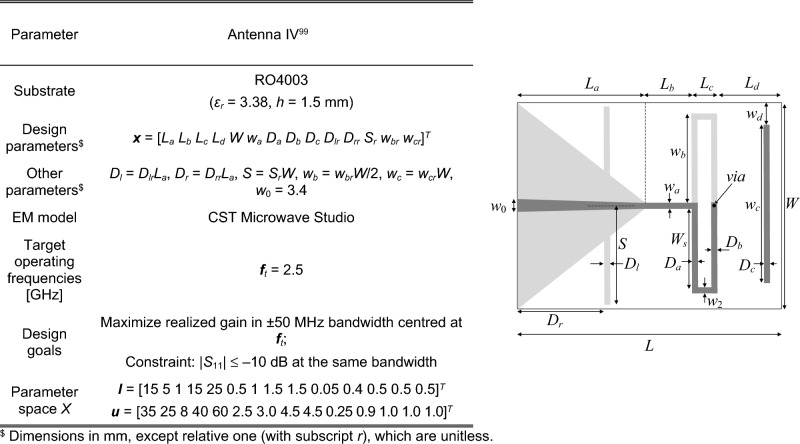
Antenna IV^99^: quasi-Yagi antenna, the light-shade grey marks ground-plane metallization; parameters (left), and antenna geometry (right).

The antennas employed for verification purposes have been presented in Figs. 8, 9, 10 and 11, where also all important details on antenna structures have been gathered. The benchmark antennas are: (i) Antenna I: a dual-band uniplanar dipole antenna, (ii) Antenna II: a triple-band dipole antenna, (iii) Antenna III: a triple-band U-slotted patch antenna with defected ground structure (DSG), (iv) Antenna IV: a quasi-Yagi antenna. For all structures, the performance vectors contain antenna reflection coefficients at the resonant frequencies, whereas, for Antenna IV, it is also the maximum gain (cf. Fig. 4). For all antennas, the EM models are simulated with the use of the time-domain solver of CST Microwave Studio. Observe that the parameter spaces are extensive for Antennas I through IV, both in terms of dimensionality, and, most importantly, with respect to the parameter ranges. The average ratio of the upper to lower bounds equals 4.2 for Antenna I, 8.5 for Antenna II, 2.6 for Antenna III, and 3.5 for Antenna IV.

### Experimental setup

Each of the antennas described in "Verification antenna structures" section has been optimized using the algorithm developed in this work. The control parameters of our framework have been set to *F*_max_ = 0.2 GHz, *α* = 0.2, *γ* = 0.5, *D*_min_ = 1, and *ε* = 10^–3^ (cf. "Optimization procedure" section for a description of these parameters). Note that the control parameter setup is identical for all antennas, which is to show that the specific values are not critical, and the algorithm does not require careful tuning before it can be successfully employed.

The Antennas I through IV were also optimized using three benchmark procedures:

- Particle swarm optimizer (PSO)^95^; the following setup is used: swarm size equals 10, maximum number of iterations 50 and 100 (Versions I and II, respectively), the remaining control parameters are set up to: *χ* = 0.73, *c*_1_ = *c*_2_ = 2.05;
- Machine learning framework with the following setup: kriging interpolation model employed as a surrogate model with the initial surrogate ensuring relative RMS error below 20% (maximum number of training samples equal to 400); minimization of the predicted objective function utilized as an infill criterion^101^;
- Trust-region algorithm with numerical derivatives (see "Local tuning using gradient-based trust-region search" section) with antenna sensitivities estimated using finite differentiation. Each run is carried out from a random starting point.

The PSO algorithm has been selected as a representative nature-inspired global optimization procedure. Note that the computational budget has been set at relatively low values (500 EM simulations for Version I, and 1000 for Version II). This is to avoid excessive costs, although one thousand EM analyses is already beyond borderline in terms of practical utility. Machine learning technique has been chosen because it allows for illustrating the typical challenges of surrogate-assisted frameworks when applied to demanding design scenarios, i.e., modeling of nonlinear antenna characteristics within multi-dimensional design spaces and wide parameter ranges. Local optimization has been included into the benchmark set to show the importance for global optimization in the case of the considered test problems.

### Results and discussion

**Figure 12:**
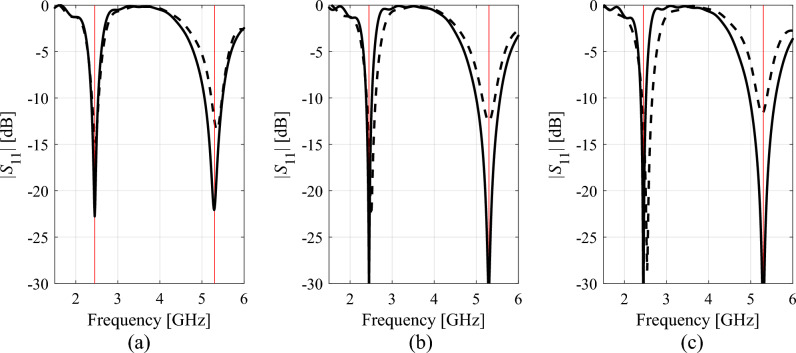
Selected Antenna I responses for the designs rendered by the developed globalized simplex-based optimization algorithm: (**a**) design 1, (**b**) design 2, (**c**) design 3. Antenna response for the initial design ***x***^(0)^ (produced by the global search stage) is shown using dashed line. Antenna response for the final design is shown by solid line. The intended centre frequencies (i.e., 2.45 and 5.3 GHz) are shown using vertical lines.

**Figure 13:**
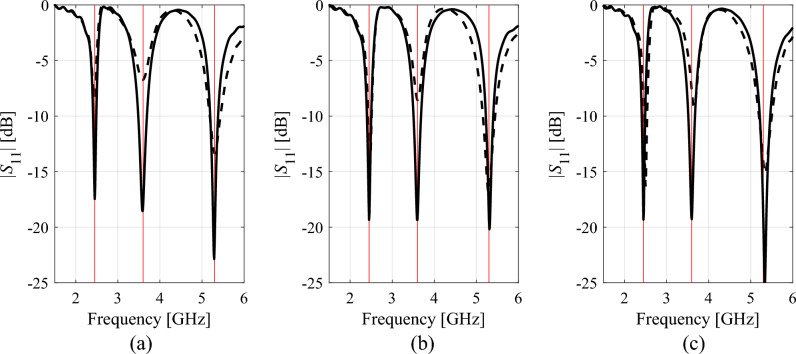
Selected Antenna II responses for the designs rendered by the developed globalized simplex-based optimization algorithm: (**a**) design 1, (**b**) design 2, (**c**) design 3. Antenna response for the initial design ***x***^(0)^ (produced by the global search stage) is shown using dashed line. Antenna response for the final design is shown by solid line. The intended centre frequencies (i.e., 2.45 and 5.3 GHz) are shown using vertical lines.

**Figure 14:**
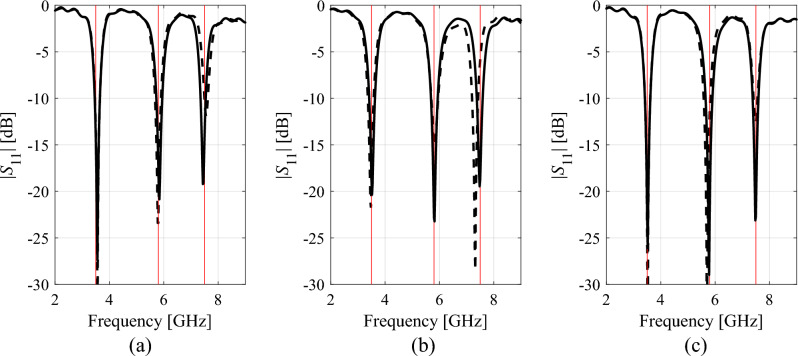
Selected Antenna III responses for the designs rendered by the developed globalized simplex-based optimization algorithm: (**a**) design 1, (**b**) design 2, (**c**) design 3. Antenna response for the initial design ***x***^(0)^ (produced by the global search stage) is shown using dashed line. Antenna response for the final design is shown by solid line. The intended centre frequencies (i.e., 2.45, 3.6, and 5.3 GHz) are shown using vertical lines.

**Figure 15:**
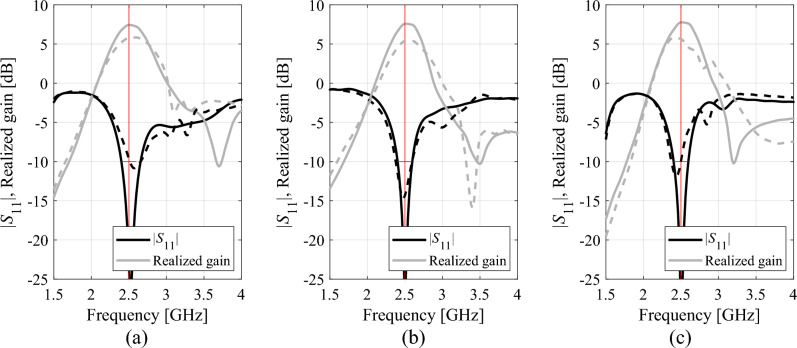
Selected Antenna IV responses for the designs rendered by the developed globalized simplex-based optimization algorithm: (**a**) design 1, (**b**) design 2, (**c**) design 3. Antenna response for the initial design ***x***^(0)^ (produced by the global search stage) is shown using dashed line. Antenna response for the final design is shown by solid line. The intended centre frequency 2.5 GHz is shown using vertical line, the target impedance bandwidth (at the level of − 10 dB) is marked using the horizontal line.

Tables 1, 2, 3, 4 present the results rendered by the proposed and the benchmark methods. Meanwhile, Figs. 12, 13, 14 and 15 show the antenna responses for the selected runs of the algorithm of "Global optimization of antennas using simplex-based predictors" section. In the course of our experiments, each algorithm was executed ten times, which was done in order to reduce a possible bias due to stochastic components within the considered procedures. The values reported in the tables refer to the average performance, including the objective function value, CPU cost, and success rate. The latter is a number of algorithm executions with the antenna operating parameters allocated sufficiently close to their target values, i.e., the condition ||***f***(x^*^) – ***f***_*t*_||< *F*_max_ was satisfied. Below, we analyze the results from the perspective of computational efficacy of the respective optimization algorithms, their reliability, as well as design quality.

**Table 1:** Antenna I: Optimization results and cost breakdown.

Optimization method	Simplex-based algorithm (this work)	PSO		Machine learning	Trust-region gradient-based algorithm
		50 iterations	100 iterations		
Average objective function value [dB]	− 25.3	− 18.2	− 19.3	− 20.7	− 13.5
Computational cost^a^	82.9	500	1000	457.8	84.2
Success rate^b^	10/10	9/10	10/10	10/10	6/10

^a^The cost expressed in terms of the number of EM simulations of the antenna structure under design.

^b^Number of algorithms runs at which the operating frequencies were allocated with sufficient accuracy, i.e., so that ||***f***(x*) – ***f***_*t*_||< *F*_max_.

**Table 2:** Antenna II: Optimization results and cost breakdown.

Optimization method	Inverse-surrogate-based algorithm (this work)	PSO		Machine learning	Trust-region gradient-based algorithm
		50 iterations	100 iterations		
Average objective function value [dB]	− 17.5	− 10.8	− 13.8	− 13.5	− 7.8
Computational cost^a^	154.0	500	1000	470.0	105.8
Success rate^b^	10/10	5/10	8/10	10/10	4/10

^a^The cost expressed in terms of the number of EM simulations of the antenna structure under design.

^b^Number of algorithms runs at which the operating frequencies were allocated with sufficient accuracy, i.e., so that ||***f***(x*) – ***f***_*t*_||< *F*_max_.

**Table 3:** Antenna III: Optimization results and cost breakdown.

Optimization method	Inverse-surrogate-based algorithm (this work)	PSO		Machine learning	Trust-region gradient-based algorithm
		50 iterations	100 iterations		
Average objective function value [dB]	− 17.5	− 12.3	− 14.2	− 14.2	− 12.1
Computational cost^a^	110.7	500	1000	473.0	125.4
Success rate^b^	10/10	6/10	8/10	7/10	4/10

^a^The cost expressed in terms of the number of EM simulations of the antenna structure under design.

^b^Number of algorithms runs at which the operating frequencies were allocated with sufficient accuracy, i.e., so that ||***f***(x*) – ***f***_*t*_||< *F*_max_.

**Table 4:** Antenna IV: Optimization results and cost breakdown.

Optimization method	Inverse-surrogate-based algorithm (this work)	PSO		Machine learning	Trust-region gradient-based algorithm
		50 iterations	100 iterations		
Average objective function value [dB]^a^	7.4	6.1	6.8	7.6	− 1.1
Computational cost^b^	144.3	500	1000	585.3	138.4
Success rate^c^	10/10	9/10	10/10	10/10	1/10

^a^The values reported in the table refer to the realized gain at the target operating frequency of 2.5 GHz.

^b^The cost expressed in terms of the number of EM simulations of the antenna structure under design.

^c^Number of algorithms runs at which the operating frequencies were allocated with sufficient accuracy, i.e., so that ||***f***(x*) – ***f***_*t*_||< *F*_max_.

It can be observed that the proposed procedure ensures perfect success rate for all verification antennas, i.e., satisfactory design was found in all of its runs. This can be considered an evidence for the global search capabilities of the algorithm. At the same time, the success rate of multiple-start local optimization is considerably worse (the average of about 4/10 across the four antenna structures), which indicates that the design problems are indeed multi-modal. As far as machine learning procedure is concerned, its success rate is perfect (10/10) for all test cases except Antenna III; however, the related computational expenses (primarily the initial costs of training data acquisition when constructing the kriging interpolation surrogates) are considerably higher.

Finally, the nature-inspired algorithm (here, PSO) performs considerably better, with the average success rate better than 7/10 for the computational budget of 500 EM simulations, and equal to 9/10 for the budget of 1000 simulations. This indicates that PSO is capable of identifying satisfactory designs, and it is likely that the success rate would become perfect is the budget is increased further. Yet, this is not a practical option due to excessive computational expenses. Notwithstanding, the average quality of the designs produced by all benchmark techniques is inferior in comparison with those produced by the proposed technique.

Computational efficacy of the developed algorithm can be assessed as excellent. Given its global search capability, the average number of EM simulations necessary to conclude the optimization process is only about 120 across the set of four antenna structures. This cost is only somewhat higher than that of the gradient search (the average of 114 EM analyzes), and dramatically lower than the expenses incurred by PSO. From the point of view of practical utility, this is one of the most important advantages of the presented procedure. As mentioned before, this has been made possible by exploiting problem-specific knowledge embedded in antenna responses and incorporating it to construct the simplex-based inverse models defined over the operating and performance figures of the structure at hand. Another advantage of our approach is that all the basic steps are fully automated, i.e., no designer’s interaction is required apart from providing the initial data.

The properties of the presented optimization algorithm make it a potentially attractive solution for quasi-global antenna optimization, which might be preferred over more conventional approaches, including surrogate-assisted procedures of the EGO type (efficient global optimization^100^) and similar, let alone nature-inspired routines. It should be mentioned that a potential limitation of our method is related to the size of the design space, especially with respect to the parameter ranges. More specifically, if the space is excessively large, the computational cost of identifying a useful set of trial points (i.e., those that are accepted in the sense discussed in "Simplex-based predictors" section) may be high because most of random observables will likely be rejected. On the other hand, if the designer is able to establish reasonable parameter bounds based on the engineering insight, this worse-case scenario would never occur. As a matter of fact, the design spaces considered for Antennas I through IV are already large: the upper-to-lower parameter bound are 4.2, 8.4, 2.5, and 3.4 (on average) for Antennas I through IV, respectively.

## Conclusion

In this work, we proposed a novel framework for quasi-global parameter tuning of antenna structures. The presented methodology relies on knowledge-based simplex-like predictors built using an automated procedure at the level of operating conditions of the antenna at hand, which effectively act as inverse models directly producing geometry parameter vectors corresponding to presumably better designs. The simplex updating scheme is developed to facilitate parameter space exploration, as well as to guarantee convergence of the process. The global search stage is supplemented by a cost-efficient local tuning that employs gradient-based algorithm with sparse sensitivity updates. Our optimization framework has been comprehensively validated using four antenna structures with their design tasks being all multimodal problems. The obtained results indicate a perfect success rate, i.e., the ability of identifying satisfactory design for all procedure runs executed during the numerical experiments. The benchmark methods, including nature-inspired algorithms (here, PSO), machine learning technique, and multiple start gradient search, exhibit significantly worse performance in terms of repeatability of solutions, and the likelihood of yielding designs that meet the assumed specifications. Furthermore, the overall design quality, measured as the value of the objective function, is superior for the proposed approach. As far as computational efficiency is concerned, it is comparable to local optimization, and minor in comparison to the nature-inspired methods.

A potential limitation of the presented optimization procedure is that is relies on proper extraction of the operating parameters of the antenna at hand from its EM simulation results, which may turn problematic for heavily distorted responses. On the other hand, the initial stage of the search process (random observable generation) already implements a safeguard by rejecting the samples for which such an extraction is not possible. Also, if the parameter space is determined using engineering insight about the considered antenna structures, the likelihood of the aforementioned issue to occur is considerably limited. It seems that the proposed technique may turn a reliable and low-cost alternative to existing global optimization routines, especially population-based methods.

## Data Availability

The datasets generated during and/or analysed during the current study are available from the corresponding author on reasonable request.
